# Association of Periodontitis with Atherosclerotic Cardiovascular Diseases: A Nationwide Population-based Retrospective Matched Cohort Study

**DOI:** 10.3390/ijerph17197261

**Published:** 2020-10-04

**Authors:** Min-Ji Byon, Se-Yeon Kim, Ji-Soo Kim, Han-Na Kim, Jin-Bom Kim

**Affiliations:** 1 Department of Preventive and Community Dentistry, School of Dentistry, Pusan National University, 49 Busandaehak-ro, Mulgeum-eup, Yangsan 50612, Korea; kyura2@naver.com (M.-J.B.); secan00@naver.com (S.-Y.K.); psily1@naver.com (J.-S.K.); 2 BK21 FOUR project, School of Dentistry, Pusan National University, Yangsan 50612, Korea; 3 Department of Dental Hygiene, College of Health and Medical Sciences, Cheongju University, Cheongju 28503, Korea; hnkim@cju.ac.kr

**Keywords:** atherosclerosis, cardiovascular disease, cohort study, heart disease, national health insurance service, periodontitis, risk

## Abstract

We investigated the association between periodontitis and atherosclerotic cardiovascular disease (ACVD) development using the National Health Insurance Service—National Sample Cohort 2.0 (NHIS-NSC2) database, which contains data for approximately 1 million nationally representative random participants. We selected 52,425 participants aged 20+ years and diagnosed with periodontitis from January to December 2003 and used propensity score matching to select an equivalent number of participants who were never diagnosed with periodontitis in the period covered by the NHIS-NSC2 database (2002–2015). The propensity scores were based on sex, age group, type of national health insurance, household income, diabetes status, and hypertension status and were used for 1:1 matching of individuals with similar propensities. A total of 104,850 participants were selected for the study. A multivariable Cox proportional hazard regression model was used to investigate the risk of ACVD development due to periodontitis from 2003 to 2015 after adjusting for sex, age, type of national health insurance, household income, hypertension status, and diabetes status. Participants with periodontitis had a higher risk of ACVD (adjusted hazard ratio: 1.09, 95% confidence interval: 1.05–1.13) than those without periodontitis. Thus, periodontitis can increase the risk of ACVD, and prevention of periodontitis may help reduce the risk of cardiovascular disease.

## 1. Introduction

Atherosclerotic cardiovascular diseases (ACVDs) are a group of diseases that include fatal and non-fatal coronary heart disease (angina, myocardial infarction), ischemic cerebrovascular disease (stroke/transient ischemic attack), and peripheral arterial disease [1]. Cardiovascular disease remains a leading cause of death worldwide, and the number of patients with cardiovascular disease has increased over the years [2,3]. The causes of ACVD are very complex, but ongoing studies have suggested that periodontal disease-related microorganisms may invade the cardiovascular system and promote or exacerbate atherosclerosis. For example, ApoE-null mice that received *Porphyromonas gingivalis* showed atherosclerosis [4]. Similarly, periodontal pathogens were detected in the tissues of patients undergoing carotid endarterectomy [5]. 

Periodontal disease is a chronic inflammatory disease caused by bacterial infection of the supporting tissues around the teeth [6]. In addition to causing oral cavity diseases, bacteria in the oral cavity are also reported to be closely related to the development of various systemic diseases. Poor oral health and tooth loss are significantly related to the development of coronary atherosclerotic burden [7]. Once periodontal disease is established, bacteria and their products enter the blood stream, activating the host inflammatory response. This is known to encourage atheroma formation, maturation, and vulnerability [1].

Dietrich et al. [8] conducted a systematic review of epidemiological studies and reported that people with periodontal disease have an increased risk of developing ACVD. However, their study was limited by the inconsistent approaches used for measurement of periodontal disease and the lack of generalizability to the entire population. The National Health Insurance Service (NHIS) released a large-scale database from 2014, in which doctors’ diagnoses were reported as research data, and this made it possible to generalize the results of the research to the entire population. However, few studies have used the NHIS database to evaluate the relationship between periodontal disease and cardiovascular disease. 

In this study, we investigated the association between periodontitis and ACVD development by using the National Health Insurance Service—National Sample Cohort 2.0 (NHIS-NSC2) database from 2002 to 2015 in South Korea.

## 2. Materials and Methods 

### 2.1. Study Population and Design

This study used the NHIS-NSC2 database from 2002 to 2015, the data for which were released by NHIS in 2017. These data were obtained from 1,000,000 nationally representative random participants, amounting to approximately 2.2% of the entire population in the NHIS in 2006. This database included all medical claims filed from January 2002 to December 2015.

**Figure 1:**
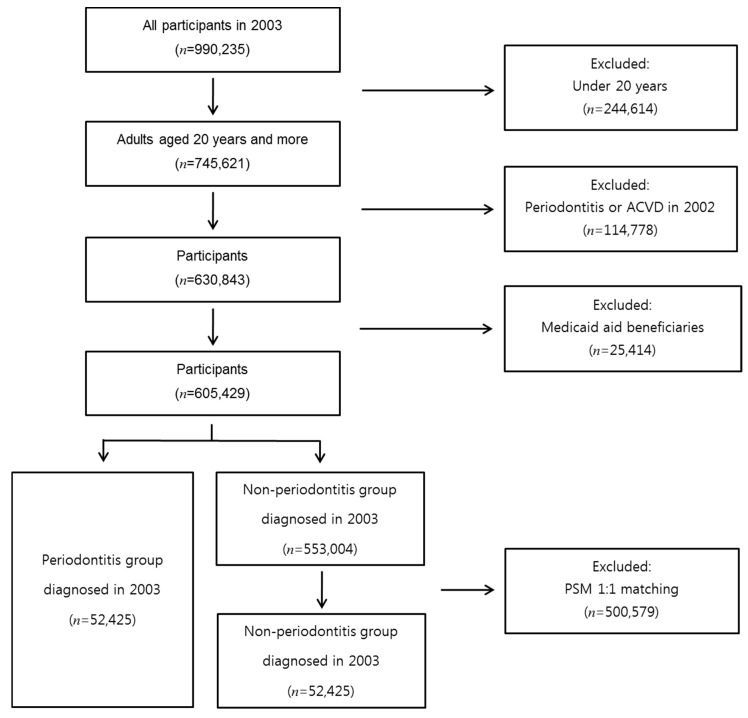
Study population selection process from the NHIS-NSC2 database. ACVD: atherosclerotic cardiovascular disease. PSM: propensity score matching. NHIS-NSC2 database: National Health Insurance Service—National Sample Cohort 2.0 database.

Among the 990,235 individuals who participated in the NHIS in 2003, 745,621 were aged 20 years or more. We excluded participants diagnosed with periodontitis or ACVD in 2002 to ensure that only participants with new episodes of periodontitis were included in the study population. We excluded medical-aid beneficiaries since the details of medical treatments for these individuals have been surveyed only since 2006. Thus, we selected a total of 52,425 participants diagnosed with periodontitis in the NHIS-NSC2 database from January to December 2003 for this retrospective cohort study. We then used propensity score matching (PSM) to select an equivalent number of participants who were never diagnosed with periodontitis during the period for which the NHIS-NSC2 database was active (2002–2015). The propensity scores were based on sex, age group, type of national health insurance, household income, diabetes status, hypertension status and were used for 1:1 matching with individuals with similar propensities. A total of 104,850 participants were selected for the study (Figure 1).

The Pusan National University Institutional Review Board judged this study to be exempt from review. The Board also waived the requirement for consent (IRB number PNU IRB/2019_98_HR).

### 2.2. Assessment of Periodontitis and ACVD

Diseases, including periodontitis and ACVD, were diagnosed using the Korean Classification of Diseases, 7th revision (KCD-7), which is a modified version of the International Classification of Disease, 10th revision (ICD-10). The NHIS uses KCD-7.

The periodontitis group included all patients diagnosed under the following KCD-7 codes one or more times in 2003: acute periodontitis (K052), chronic periodontitis (K053), periodontosis (K054), other periodontal disease (K055), and unspecified periodontal disease (K056) (Table 1). Participants newly diagnosed with periodontitis after 2004 were excluded from the study.

**Figure 2:**
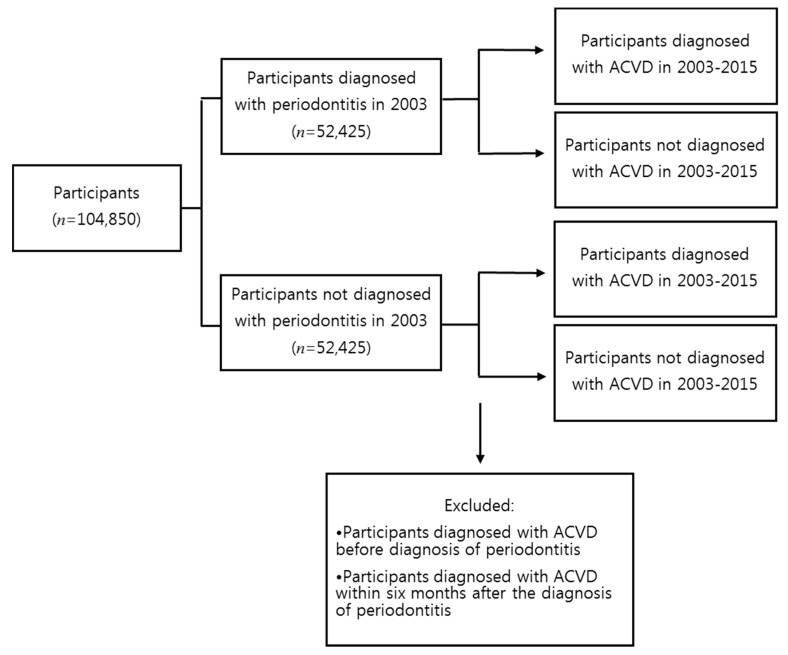
Process of selecting participants diagnosed with ACVD. ACVD: atherosclerotic cardiovascular disease.

ACVD was defined by the following KCD-7 codes: atherosclerosis (I70), cerebral atherosclerosis (I67.2), atherosclerotic cardiovascular disease, so described (I25.0), and atherosclerotic heart disease (I25.1) (Table 1). The study included participants diagnosed with ACVD from 2003 to 2015, except those treated with the above codes in 2002. Participants diagnosed with ACVD before diagnosis of periodontitis and those diagnosed with ACVD within six months of the diagnosis of periodontitis were excluded to limit the cases to those that potentially showed ACVD development due to periodontitis (Figure 2).

### 2.3. Risk Factors for ACVD 

The regression models were adjusted for the participant’s sex, age (20–29, 30–39, 40–49, 50–59, ≥60 years), type of national health insurance (self-employed insured, householder of self-employed insured, employed insured, dependents of insured employee), household income, hypertension status, and diabetes status. Household income was classified in the first to fifth quintiles. The first quintile represented the lowest household income group. Hypertension was defined using the following KCD-7 codes: essential (primary) hypertension (I10), hypertensive heart disease (I11), hypertensive renal disease (I12), hypertensive heart and renal disease (I13), and secondary hypertension (I15). Diabetes mellitus was defined using the following KCD-7 codes: type 1 diabetes mellitus (E10), type 2 diabetes mellitus (E11), malnutrition-related diabetes mellitus (E12), other specified diabetes mellitus (E13), and unspecified diabetes mellitus (E14) (Table 1). 

### 2.4. Statistical Analysis

Chi-squared tests were performed to identify the socioeconomic and systemic status of the participants. To identify the risk of ACVD development due to the prevalence of periodontitis, hazard ratios (HRs) and 95% confidence intervals (CIs) were calculated via Cox proportional hazard regression analysis. The survival rate was calculated using the Kaplan–Meier curve for the 12.5-year (150-month) follow-up period, and the log-rank test was performed to examine the differences in ACVD development between the periodontitis and non-periodontitis groups. Statistical significance was set at 0.05. Analyses were conducted with SAS Enterprise Guide 7.1 (SAS Institute Inc., Cary, NC, USA) and R studio version 3.3.3 (R studio Inc., Boston, MA, USA).

## 3. Results

### 3.1. Socioeconomic Status of the Participants

Of the 104,850 study participants, 52,425 were in the periodontitis group and 52,425 in the non-periodontitis group. Both groups contained 50.3% males and 49.7% females. No significant intergroup difference was observed in sex, age group, type of national health insurance, household income, hypertension, and diabetes mellitus, since these variables had been used for propensity score matching. The prevalence of ACVD due to periodontitis was significantly different in the two groups: 11.2% in the periodontitis group and 10.5% in the non-periodontitis group (Table 2). 

### 3.2. Risk Factors for ACVD

A Cox proportional hazard regression model was used to investigate the risk factors for ACVD development. In the univariable Cox proportional hazard regression model, the risk of ACVD in males (HR: 1.09, 95% CI: 1.05–1.13) was higher than that in females. The age-specific HR of ACVD increased with age from 3.46 (30–39 years) to 25.67 (60–69 years). With regard to the type of national health insurance, the risk of ACVD in householder of self-employed insured (HR: 1.54, 95% CI: 1.46–1.62) and in employed insured (HR: 1.58, 95% CI: 1.50–1.67) was higher than that in self-employed insured. With respect to household income, the first quintile of participants with the lowest household income showed the highest risk of ACVD (second quintile: HR: 0.77, 95% CI: 0.73–0.81; third quintile: HR: 0.74, 95% CI: 0.70–0.78; fourth quintile: HR: 0.69, 95% CI: 0.65–0.73; fifth quintile: HR: 0.81, 95% CI: 0.77–0.86). Participants with hypertension showed a higher risk of ACVD (HR: 8.16, 95% CI: 7.74–8.61), as did participants with diabetes mellitus (HR: 5.02, 95% CI: 4.81–5.25) and those with periodontitis (HR: 1.08, 95% CI: 1.04–1.12).

In the multivariable Cox proportional hazard regression model adjusted for all variables (sex, age group, type of national health insurance, household income, hypertension status, diabetes mellitus status, periodontitis status), the age-specific adjusted HR (aHR) of ACVD increased with age from 2.27 (30–39 years) to 6.29 (60–69 years). For household income, the first quintile of participants with the lowest household income showed the highest risk of ACVD (second quintile: aHR: 0.90, 95% CI: 0.86–0.95; third quintile: aHR: 0.90, 95% CI: 0.85–0.95; fourth quintile: aHR: 0.87, 95% CI: 0.82–0.92; fifth quintile; aHR: 0.93, 95% CI: 0.87–0.98). Participants with hypertension had a higher risk of ACVD (aHR: 3.64, 95% CI: 3.44–3.86), as did participants with diabetes mellitus (aHR: 2.18, 95% CI: 2.08–2.28) and those with periodontitis (aHR: 1.09, 95% CI: 1.05–1.13) (Table 3).

### 3.3. Association of ACVD with Periodontitis

**Figure 3:**
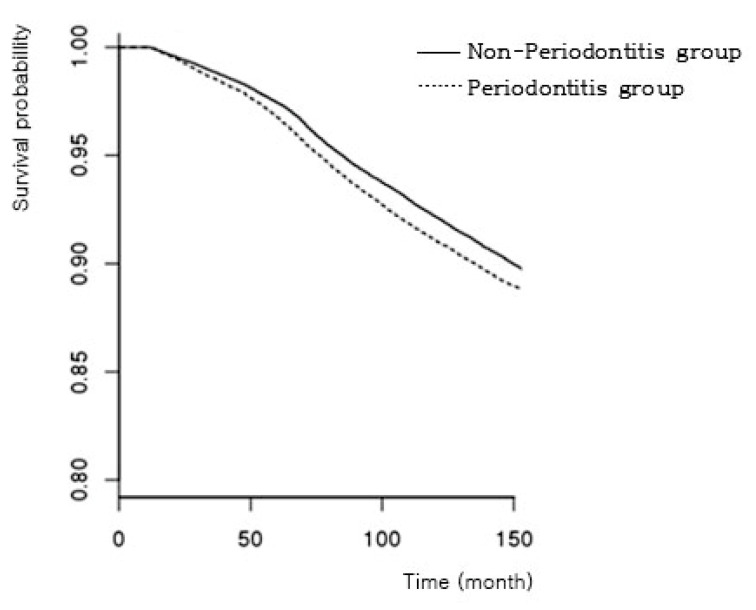
Kaplan–Meier curves for the incidence of ACVD by the presence of periodontitis.

The survival rate was calculated using the Kaplan–Meier curve for the 12.5-year (150-month) follow-up period, and the log-rank test was performed to examine differences in ACVD development between the periodontitis and non-periodontitis groups. The incidence of ACVD in participants with periodontitis was higher than that in participants without periodontitis (*p* < 0.001) (Figure 3). The survival rate indicating no development of ACVD for 150 months was 88.83% in the periodontitis group and 89.17% in non-periodontitis group.

## 4. Discussion

We examined 104,850 participants over 13 years in this retrospective matched cohort study. The participants were extracted from a national database of 990,235 randomly selected participants and matched on the basis of socioeconomic and systemic health status. We found that participants with periodontitis exhibited a higher prospective risk for ACVD development during a 13-year follow-up period after adjusting for sex, age group, type of national health insurance, household income, hypertension status, and diabetes mellitus status. In the periodontitis group, the incidence of ACVD was 11.2%, which was higher than the 10.5% incidence in the non-periodontitis group. The aHR of ACVD was 1.09 (95% CI: 1.05–1.13) in the periodontitis group.

Infection by oral bacteria causes inflammation throughout the whole body, such as periodontal tissues and vascular endothelium, and induces atherosclerotic responses of varying degrees depending on the immunity of the host [9]. *P. gingivalis* increases morbidity in periodontal disease in the oral cavity and induces chronic inflammation in the periodontal tissue [10]. The inflammatory cytokines tumor necrosis factor-α, interleukins 6 and 8, and C-reactive protein are expressed in high concentrations in the saliva or gingival effusion because of local inflammatory reactions in the oral cavity. These substances have been reported to induce a systemic inflammatory reaction by penetrating and circulating through the vascular system [11]. In periodontitis patients, the subgingival microbial burden contributes to endotoxemia. Lipopolysaccharide is a possible molecular mediator between periodontal disease and coronary artery disease [12]. The DNA of *P. gingivalis* has been confirmed to be detected in vascular endothelial cell atheroma, confirming that the bacteria involved in pathological periodontal disease are closely linked to atherosclerosis [13].

Periodontitis is a substantially important risk factor for atherosclerotic vascular disease. Periodontitis was associated with both subclinical atherosclerosis (adjusted odds ratio (aOR): 1.55, 95% CI: 1.07–2.24) and peripheral arterial disease (aOR: 2.03, 95% CI: 1.05–3.93) in Korean adults [14]. Periodontitis was also independently associated with ACVD (OR: 1.59, 95% CI: 1.39–1.81) in the Netherlands [15]. Similarly, patients with chronic apical periodontitis in Brazil showed a 2.79-fold higher risk of developing coronary artery disease [16].

In the retrospective cohort study using the NHIS database, the HR of peripheral arterial disease in the periodontitis group was 1.15 (95% CI: 1.07–1.23) in comparison with that in the matched group [17]. In a Danish nationwide cohort study, the incidence rate ratio (IRR) of myocardial infarction in the periodontitis group was 1.16 (95% CI: 1.04–1.30), and the IRR of cardiovascular death in the periodontitis group was 2.02 (95% CI: 1.87–2.18), in comparison with that in the control group [18].

As such, this cohort study confirmed that periodontitis can be a risk factor for ACVD (aHR: 1.09, 95% CI: 1.05–1.13). Therefore, the risk of ACVD should be considered when treating or managing patients with periodontitis. Good oral hygiene is recommended to reduce cardiometabolic disease risk through the prevention of periodontitis [19]. Counselling on balanced dietary habits and avoiding smoking are recommended to improve resistance to dental caries or periodontitis, which may improve general health and possibly also improve the risk factors for cardiovascular disease [20]. Periodontitis, which is one of the causes of ACVD, should not be taken lightly, but it is easy to treat the initial gingivitis.

This study had some limitations. Most dentists working at dental clinics in Korea did not undergo calibration training to apply the same diagnostic criteria in the diagnosis of periodontitis. They do not actively diagnose and treat periodontitis since they rely on visual examinations to diagnose periodontitis. Moreover, the reimbursement of periodontitis treatment in some cases may have been rejected by the Health Insurance Review and Assessment Service related to NHIS if there were mistakes in the application process for treatment reimbursement. Therefore, it is possible that some participants were classified as “non-periodontitis”, instead of being classified as participants with periodontitis. Consequently, the aHR for the risk factor of ACVD in this study may have been underestimated.

This nationwide investigation of the association between periodontitis and ACVD is one of the largest of its kind to date. It has the advantage that it can be generalized using data from a nationwide retrospective matched cohort study with a large sample. Unlike previous studies that only confirmed the relationship between periodontitis and cardiovascular disease using cross-sectional studies, this study confirmed that periodontitis is related to ACVD development.

## 5. Conclusions

This study investigated the association between periodontitis and ACVD development using the National Health Insurance Service—National Sample Cohort 2.0 (NHIS-NSC2) database. The results showed that participants with periodontitis had a 9% higher risk of ACVD. Our findings confirmed that periodontitis can increase the risk of ACVD development, and it is important to prevent and manage periodontitis to improve systemic health and possibly also reduce the risk for cardiovascular disease.

## Figures and Tables

**Table 1:** KCD-7 codes used in disease definition.

Disease	Diagnosis	KCD-7 Code
Periodontitis	Acute periodontitis	K05.2
	Chronic periodontitis	K05.3
	Periodontosis	K05.4
	Other periodontal diseases	K05.5
	Periodontal disease, unspecified	K05.6
Atherosclerotic cardiovascular disease (ACVD)	Atherosclerosis	I70
	Cerebral atherosclerosis	I67.2
	Atherosclerotic cardiovascular disease, so described	I25.0
	Atherosclerotic heart disease	I25.1
Hypertension	Essential (primary) hypertension	I10
	Hypertensive heart disease	I11
	Hypertensive renal disease	I12
	Hypertensive heart and renal disease	I13
	Secondary hypertension	I15
Diabetes mellitus	Type 1 diabetes mellitus	E10
	Type 2 diabetes mellitus	E11
	Malnutrition-related diabetes mellitus	E12
	Other specified diabetes mellitus	E13
	Unspecified diabetes mellitus	E14
KCD-7: Korean Classification of Diseases, 7th revision.		

**Table 2:** Socioeconomic and systemic status of the participants by diagnosis of periodontitis.

Variables	Non-periodontitis Group No. (%)	Periodontitis Group No. (%)	*p*-Value
Total	52,425 (50.0)	52,425 (50.0)	
Sex			1.00
Male	26,345 (50.3)	26,345 (50.3)	
Female	26,080 (49.7)	26,080 (49.7)	
Age group (years)			1.00
20–29	8566 (16.3)	8566 (16.3)	
30–39	11,376 (21.7)	11,376 (21.7)	
40–49	13,667 (26.1)	13,667 (26.1)	
50–59	9578 (18.3)	9578 (18.3)	
≥60	9238 (17.6)	9238 (17.6)	
Type of national health insurance			1.00
Self-employed insured	13,268 (25.3)	13,268 (25.3)	
Householder of self-employed insured	12,122 (23.1)	12,122 (23.1)	
Employed insured	12,901 (24.6)	12,901 (24.6)	
Dependents of insured employee	14,134 (27.0)	14,134 (27.0)	
Household income (%)			1.00
First quintile (lowest)	14,465 (27.6)	14,465 (27.6)	
Second quintile	12,199 (23.3)	12,199 (23.3)	
Third quintile	10,970 (20.9)	10,970 (20.9)	
Fourth quintile (highest)	7923 (15.1)	7923 (15.1)	
Fifth quintile	6868 (13.1)	6868 (13.1)	
Hypertension			1.00
No	28,520 (54.4)	28,520 (54.4)	
Yes	23,905 (45.6)	23,905 (45.6)	
Diabetes mellitus			1.00
No	29,908 (57.1)	29,908 (57.1)	
Yes	22,517 (42.9)	22,517 (42.9)	
ACVD			<0.001
No	46,928 (89.5)	46,568 (88.8)	
Yes	5497 (10.5)	5857 (11.2)	

ACVD: atherosclerotic cardiovascular disease. *p*-values were obtained by chi-squared tests.

**Table 3:** Cox proportional hazard regression model for the risk factors of ACVD.

Variables	Univariable Cox Regression		Multivariable Cox Regression *	
	HR	95% CI	Adjusted HR	95% CI
Sex				
Female (ref)	1		1	
Male	1.09	1.05–1.13	0.99	0.95–1.04
Age group				
20–29 (ref)	1		1	
30–39	3.46	2.94–4.07	2.27	1.93–2.68
40–49	9.60	8.24–11.18	3.99	3.41–4.66
50–59	19.29	16.59–22.43	5.75	4.92–6.72
≥60	25.67	22.09–29.83	6.29	5.38–7.36
Type of national health insurance				
Self-employed insured (ref)	1		1	
Householder of self-employed insured	1.54	1.46–1.62	0.92	0.91–1.03
Employed insured	1.58	1.50–1.67	0.98	0.93–1.04
Dependents of insured employee	1.02	0.96–1.09	0.95	0.88–1.01
Household income (%)				
First quintile (lowest) (ref)	1		1	
Second quintile	0.77	0.73–0.81	0.90	0.86–0.95
Third quintile	0.74	0.70–0.78	0.90	0.85–0.95
Fourth quintile	0.69	0.65–0.73	0.87	0.82–0.92
Fifth quintile (highest)	0.81	0.77–0.86	0.93	0.87–0.98
Hypertension				
No (ref)	1		1	
Yes	8.16	7.74–8.61	3.64	3.44–3.86
Diabetes mellitus				
No (ref)	1		1	
Yes	5.02	4.81–5.25	2.18	2.08–2.28
Periodontitis				
No (ref)	1		1	
Yes	1.08	1.04–1.12	1.09	1.05–1.13

ACVD: atherosclerotic cardiovascular disease. HR: hazard ratio. CI: confidence interval. * Multivariable Cox proportional hazard regression model was adjusted for sex, age group, type of national health insurance, household income, hypertension status, diabetes mellitus status, and periodontitis status.
